# The experience of feeling old after a fragility fracture

**DOI:** 10.1186/s12877-024-04769-w

**Published:** 2024-02-22

**Authors:** Joanna E.M. Sale, Lucy Frankel, Earl Bogoch, Gabriel Carlin-Coleman, Sean Hui, Jessica Saini, Jennifer McKinlay, Lynn Meadows

**Affiliations:** 1 Musculoskeletal Health and Outcomes Research, Li Ka Shing Knowledge Institute, St. Michael’s Hospital, Unity Health Toronto, 30 Bond Street, M5B 1W8 Toronto, ON Canada; 2 Institute of Health Policy, Management and Evaluation , University of Toronto, 4th Floor– 155 College Street, M5T 3M6 Toronto, ON Canada; 3 Department of Surgery, Faculty of Medicine, University of Toronto, 5th Floor– 149 College Street, M5T 1P5 Toronto, ON Canada; 4 Department of Surgery, University of Toronto, St. Michael’s Hospital, Unity Health Toronto, 30 Bond Street, M5B 1W85 Toronto, ON Canada; 5 Brookfield Chair in Fracture Prevention, University of Toronto, St. Michael’s Hospital, Unity Health Toronto, 30 Bond Street, M5B 1W8 Toronto, ON Canada; 6 Department of Community Health Sciences , University of Calgary, 3D10 - 3280 Hospital Drive NW, AB T2N 4Z6 Calgary, Canada

**Keywords:** Fragility fracture, Subjective age, Qualitative research, Patient perspective, Bone health

## Abstract

**Background:**

There has been little exploration of the effect of fragility fractures on patient perceptions of their age. The common assumption is that fractures “happen to old people”. In individuals with a fragility fracture, our objective was to explore the experience of feeling old after sustaining a fragility fracture.

**Methods:**

A secondary analysis of data from 145 community-dwelling women and men participating in six qualitative primary studies was conducted relying on a phenomenological approach. Participants were English-speaking, 45 years and older, who had sustained a recent fragility fracture or reported a history of previous fragility fractures. Data for the analysis included direct statements about feeling old as well any discussions relevant to age post-fracture.

**Results:**

We highlight two interpretations based on how individuals with a history of fragility fracture talked about age: (1) Participants described feeling old post-fracture. Several participants made explicit statements about being “old”. However, the majority of participants discussed experiences post-fracture that implied that they *felt* old and had resigned themselves to *being* old. This appeared to entail a shift in thinking and perception of self that was permanent and had become a part of their identity; and (2) Perceptions of increasing age after sustaining a fracture were reinforced by health care providers, family, and friends.

**Conclusions:**

Our findings challenge the notion that fractures “happen to old people” and suggest that fractures can make people feel old. Careful consideration of how bone health messages are communicated to patients post-fracture by health care providers is warranted. (Word Count: 248)

## Background

The association between chronological age and fragility fractures and bone health is well established. According to the World Health Organization, the vast majority of fragility (or osteoporotic) fractures occur in individuals who are “elderly” and the incidence of these fractures increase markedly with age [1]. Diagnostic testing, such as bone mineral density (BMD) testing, is recommended at 50 + years of age by the Canadian clinical practice guidelines for the diagnosis and management of osteoporosis [2]. If the individual sustains the fragility fracture after the age of 40, the BMD test is recommended after the fracture [2]. Fracture risk assessments are also dependent upon age [3, 4]. As a result, the common assumption is that fractures *happen to old people*. To illustrate, a metasynthesis of qualitative studies demonstrated that the image of osteoporosis was often one of an “old woman with a bent body and collapsing back” and that not much could be done about poor bone health because it is part of the aging process [5]. In this paper, we propose that the reverse, that fractures *make people old*, could also resonate with individuals.

There has been little exploration of the effect of fractures on subjective age. Our previous work with patients who sustained a fragility fracture appeared to challenge the notion that fractures are perceived to happen to individuals who feel “old”. We reported that patients rejected the concept of fragility fracture, implying that there was nothing fragile about the fracture which was perceived to be a physical and traumatic event [6]. However, it has been demonstrated that patients with fragility fractures report being increasingly dependent on others *after* their fracture [7] and that they have a heightened fear of falling which results in their being more careful [8]. This suggests that fragility fractures can have a profound age-related meaning to individuals, especially when they are told they are at risk for future fracture.

We set out to explore the concept that fractures make people feel old for a number of reasons. Subjective age has been shown to predict important health outcomes such as engagement in many behaviours including personal and social activities [9]. Feeling older has been shown to be associated with a considerable decrease in life satisfaction [10] and higher risk of mortality [11, 12]. Further, the belief that one’s bone health is associated with aging has also been shown to be associated with poor medication adherence to bisphosphonates [13].

The purpose of our study was to conduct a secondary analysis of qualitative data on the experience of individuals with fragility fractures to determine the meaning of these fractures based on discussions about age. Specifically, our objective was to examine the reported experience of feeling old after a fragility fracture.

## Methods

This secondary analysis was based on six phenomenological datasets (labelled AD, I, FR, COP, COM, and M) collected over a 10-year period consisting of 220 interviews with 145 individuals with a fragility fracture (118 women and 27 men aged 45 + years). Phenomenology emphasizes the importance of direct experiences, perceptions and actions [14, 15] and the outcome of analysis is a description of the essence, or structure, of what is perceived and experienced across study participants [16]. We employed semi-structured interviews with community-dwelling individuals in all primary studies either in person (5 studies) or by telephone (1 study). The purpose of each study was to: understand the experience of adherence to bone health treatment recommendations (AD); examine intentions toward bone health investigation and treatment (I); examine perceptions of future fracture risk (FR); examine the uptake of bone health investigation and treatment recommendations in an osteoporosis patient group (COP); examine how patients with multiple chronic conditions manage bone health (COM); and explore the relationship between patients’ interpretation of their bone densitometry results and perception of bone health status (M). Three studies involved one interview and three studies involved two or three interviews. Twenty-seven participants had sustained a hip fracture and 118 had sustained a non-hip fracture. Details of the dataset have been reported previously [17]. Between 2009 and 2018, participants in the primary studies were recruited from fracture clinics in Ontario that offered a fracture prevention program or Fracture Liaison Service (*n* = 5) and from members of Osteoporosis Canada’s Canadian Osteoporosis Patient Network (*n* = 1). This national patient group of people living with osteoporosis disseminates information to members about bone health and fracture prevention.

The first author (JEMS) led the conduct of all primary studies which allowed accumulated background knowledge about the data and a tacit understanding acquired by the team in “being there” [18]. However, we acknowledge that this may also have brought pre-conceived ideas and impressions of the data. For this reason, we introduced three new analysts who were not familiar with the data to the study. Having the first author as lead of both the primary studies and the current investigation enabled the team to evaluate the adequacy of the original data and the relevance of them for the new research question proposed [19]. In this re-analysis, we asked new questions of the data [18].

Relying on a phenomenological perspective by focusing on participants’ experiences, we attended to all direct and indirect discussions that appeared to be related to aging in the interview transcripts. Participants did not need to mention the word “age” or comment about getting “old” for their data to be relevant to our objective. We were particularly interested in perceived messages from health care providers and others after individuals had sustained a fracture (including advice from family and friends) as well as discussions about expectations for the future. We excluded discussions about the association between age and fracture risk as determined by fracture risk tools as well as interactions with health care providers who recommended a BMD test because of age. We considered these conversations to be justified as the Canadian clinical practice guidelines state that patients who have sustained a fragility fracture after the age of 40 should be recommended a BMD test [2] and that age is a predictor of fracture risk [3, 4]. We also excluded any discussions about increased vigilance related to fear of falling as well as increased dependence on others as we have previously published on these topics [7, 8]. Finally, discussions about the quality of one’s bones specifically in relation to fracture risk were not eligible as this has been previously reported [8].

JEMS had previously coded and analyzed all transcripts from the six primary studies and LF had previously coded and analyzed all transcripts from five of the six primary studies. All transcripts were stored in NVivo [20]. Three new analysts recoded the 220 interviews to extract and categorize data they deemed relevant to our objective. Each transcript was assigned to two of the three analysts who worked as a team to code and discuss the data. Guided by Giorgi [21], we divided the data into parts. Codes relevant to the experience of aging were created and supported with statements by participants. The team reflected on the relationships among the codes and developing themes. JEMS and LF organized the extracted data to develop three tables in Microsoft Word [22] that were the foundation for our interpretation. These tables included explicit statements by participants about feeling old, discussions that suggested participants felt old, as well as perceived messages from health care providers and family and friends about participants’ age.

We promoted rigour in several ways. The first author led each of the primary studies so we were able to ensure there was a fit between the primary data sets and the question for the secondary analysis [23] and that our interpretation was authentic [24]. Both the primary and secondary studies were conducted within the phenomenological perspective, thus promoting theoretical and methodological consistency between the primary and secondary analyses [25]. Analysts who were unfamiliar with the original datasets recoded the transcripts to allow for a fresh perspective [26]. Data that did not fit with our overall interpretation were identified as negative cases and described as part of the results [16].

## Results

The data from the primary studies were deemed adequate to address our objective. Discussions by 143 of the 145 participants appeared relevant to the experience of aging. Experiences and perceptions discussed by participants included expectations for recovery, increased vigilance, dependence on others, concern about appearing old to others, interactions with health care providers, a family history of fragility fractures, and modifications to daily behaviours. In our examination of how individuals with a history of fragility fracture talked about age, we highlight two interpretations: (1) Participants described feeling old post-fracture. A significant number of participants made explicit statements about being “old”. However, the majority of participants discussed experiences post-fracture that implied that they *felt* old and had resigned themselves to *being* old. This appeared to entail a shift in thinking and perception of self that was permanent and had become a part of their identity; and (2) Perceptions of increasing age after sustaining a fracture were reinforced by health care providers, family, and friends. There appeared to be no connection between the type of fracture and the themes described. Quotations are identified with abbreviations for the six studies as AD, I, FR, COP, COM, and M.

### Participants described feeling old post-fracture

In their discussions, participants spoke directly about feeling old after their fracture. They talked about being an “old lady” (AD-6), “getting old” (AD-20), “getting there [to old age]” (I-21), going to walk around the mall “like an old person” (I-22), and “walking around like an old woman” (I-24). They made comments such as, “67 is not young” (FR 5) and fractures were to be expected as “part of growing old” (FR 27). Several participants talked about feeling older than their actual age. According to one participant, “I feel like I’m eighty, not fifty-three” (COP-8). In response to hearing that she had the bones of a 69-year old, one participant said, “I aged very quickly” (COM-18).

Participants talked about feeling too old to do certain activities. One participant said, “not doing what you used to do, it annoys you so much” (COP-13). Another participant told us she realized that she was “too old to continue looking after her property” (AD-5).

Participants expressed concern about how their state of being “old” appeared to others. One participant said, “when you see me walking in those [icy] conditions, I’m like a little old lady” (COP-3). One participant talked about how staff in the fracture clinic thought of her as “this old lady with this old body” (COM-3). Another participant said she hoped nobody saw her shuffling along “like a hundred-year old” (M-10). One participant resisted the recommendation to use a walker because she felt “it [the walker] makes me feel ancient” (COM-8). Similarly, participants told us that using a walker or a cane was “not where I want to be right yet” because using such devices would make them feel like “giving up” (COM-12).

#### Participants described themselves as weak and fragile

Participants referred to themselves as weak and fragile, as if they were “falling apart” (COP-24). One participant compared herself to a television commercial for pain medication, “I get up and my back, I feel like that old person on TV who puts their hand on their lower back and slowly has to push themselves back up again. Where did this come from? This isn’t me” (COP-9). After sustaining several vertebral fractures, one participant said, “It’s not I’m feeling sick, I’m not dying or sick, I’m just fatigued…you’re getting older, you can’t do [anything] about it” (COP-13). After learning about her risk of sustaining future fractures, one participant said, “It made me realize my age and it’s upsetting that I could break so easily…I kind of thought I was superman in a way…it’s…made me aware of how fragile I am. Up until that night [night of fracture], I moved sofas and stuff” (FR-4). Other participants spoke about becoming “a little frailer” (AD-10) or thinking of themselves as “more fragile, less invincible” (COM-17) and several talked about their bones as being “fragile” (FR-16; FR-21; COM-16; I-21), “more brittle” (COM-20) or “crumbling away” (COP-24). Two participants said, “I will fall apart like a weak building” (FR-17) and “my bones are not going to be strong enough to support me” (COM-12).

#### The future was foreboding

Participants’ outlook for the future were not optimistic. In their discussions, many participants anticipated that something would go wrong with their health. For example, COM-3 said, “something is going to happen sometime”. COM-16 said, “I should be paying attention to the fact that…another fall might not be as good or as easy to fix” and COM-19 said, “I just worry about aging. Is it going to get to the point where I can’t care for myself?”. One participant talked about becoming “stooped over”, and said, “five years from now, go I” (AD-6). This view of the future was apparent in discussions by other participants. For example, AD-16 said, “there is no way to have as healthy bones as a young person…when you get to this age, by nature, the bone deteriorates”. Others said, “as you get older…it’s [weak bones] bound to happen to you” (AD-21), “I don’t think your bones get stronger” (M-3), and “I could have a fracture at any time” (FR-6). FR-1 remarked that at his age, “people who break their hips…are finished”.

Old age was discussed as a factor contributing to worse recovery and poor outcomes. For example, one participant said, “it takes a long time to recover [from a fracture], especially at our age” (COP-17). Participants reported needing to take preventive measures to adapt to advancing age or to mitigate further decline. One participant talked about using a walker because it was a “good idea” to use it at her age (FR-10).

#### Reference to becoming like one’s parents

Participants’ discussions were often tied to the experiences of their parents. Statements made by participants during the interviews included: “my mom is so bad and I’m very much exactly the same sort of frame” (COP-20); “my mother…has it [osteoporosis] all over” (I-21); and “my mother had…osteoporosis…my grandmother did, too…I have to really be careful of going into the future” (FR-5). One participant whose mother had sustained multiple fragility fractures wondered “if that’s what my pattern is going to be” (COM-3). I-24 compared herself to her mother who had also sustained a fracture, “everyone is telling me - ‘like mother, like daughter’”. These discussions about one’s parents sometimes appeared to reflect concerns and a heightened awareness about one’s own mortality. AD-5 told us, “my mother had osteoporosis badly when she died. So, I was used to hearing of it [compromised bone health]”. I-18 said, “my father had a hip fracture” and he “died of a heart attack 10 days after his hip fracture surgery”.

#### Mourning the loss of youth

Implications of feeling old were apparent in participants mourning the loss of youth. For example, COP-8 said, “I used to be unbelievably active”. COP-13 told us, “you’re not as fast as you used to be, your brain goes…away slowly, you know it. You’re not doing the things you used to do”. FR-14 said, “people stop me from doing things I was doing before…like jumping off a boathouse to go in the water. Everybody is yelling ‘no, don’t let her, don’t do it’”. Similarly, another participant said, “when there is something heavy, I won’t touch it anymore. Before that, I would say, ‘Let’s do it’” (FR-20). Other participants made statements, such as: “I could lift like a rack as a young guy…but now I realize that lifting a [lock] box is heavy for me” (AD-17); “I come up from the subway…and I think I am 22 years old…no wonder I was out of breath” (AD-18). One participant talked about observing younger “kids” on television doing dangerous stunts and walking away without injuries. He said, “you’re aware that bodies are only supple when they’re relatively young. These kids…that ride their bikes off…roofs…and there’s a 20 or 30-foot gap. Boom…then they get up and walk away” (COM-10). He continued, “I used to run up and down stairs like two steps at a time. Couldn’t even think of doing that now.” Similarly, one 45-year old woman said, “I used to run and skip and hop and dance with my kids…I don’t do that anymore” (COM-18). M-9 told us, “one’s reactions are not as good as these people that fly past me going down the subway stairs and I think I used to go like that once but not anymore”. I-24 lamented the fact that she could no longer wear “high heels”.

### Perceptions of increasing age after sustaining a fracture were reinforced by health care providers, family, and friends

The sentiment of feeling old after sustaining a fracture was reinforced by health care providers as well as family members and friends. Perceived messages were both spoken and unspoken.

Participants reported being told by their physician that they should exercise caution in the long-term and give up certain activities. One 63-year old participant told us, “my family doctor said, ‘Just go slowly. Don’t rush anywhere so you’re going to trip and fall’” (FR-12). Another participant said that a nurse had told them, “what happens is we have an incident and something happens, and you go down. Then, you come up again, but not quite as far. Then you…go along quite nicely for a long time. Then, you have another incident, and these little incidents gradually wear you out” (COM-3).

Participants were told that their symptoms, such as fatigue and pain, were part of aging. For example, one participant said, “I told my doctor about it [fatigue] and he said, ‘you’re getting older’” (COP-13). One 64-year old woman said that her physician said, “considering your age and your weight, you expect to have back pain” (M-13).

Participants talked about being treated differently because of their age. One participant said, “when something goes wrong like this accident [fracture], everything sort of goes into high gear around you because they [health care providers] think you’re elderly” (M-9).

Similarly, participants said they were told they would not heal as quickly as when they were younger or that their recovery post-fracture would be slow, because of their age. According to one participant, “my doctor mentioned to me [that] when you’re 72 years old, you don’t heal as fast as you do when you’re in your 20’s” (FR-1). AD-22 told us that the physician who set her wrist told her, “because of my age, he wasn’t sure that the bones were going to knit properly”.

In some cases, participants were told that at a certain age, preventative behaviours would have no effect. One participant told us that a physician had said to her “it doesn’t really seem to matter what you do…in terms of diet, medicine…especially after age 65. There’s not a lot you can do to change what’s happened [with your bone health]” (AD-6).

Participants recounted being told they had the bones of a much older person by health care providers. One participant said that he was told his bones were like the bones of a 75 year-old man although he was only 51 years old at the time (FR-22). Other participants said they were told they had “the bone density of a 69 year old female” (46 years old at the time of the interview) (COM-18) and that they had the back “of a 70-year old” (50 years old at the time of the interview) (M-16).

Participants also reported being told by health care providers that, as they got older, their bones would deteriorate and they would develop osteoporosis. For example, one participant said that a health care provider had told her “after a certain age, it [osteopenia] does set in” (AD-5) and another said she was told by her family physician, “because of my age group, I might be a candidate for osteoporosis” (AD-10).

Messages from family and friends also reinforced participants’ feelings of becoming old. This was inherent in the way family members and friends were perceived to view them, in the advice that they received, or the way they were treated by family and friends. Participants told us that family members told them, “if you fall, you know that you might not come out of hospital” (COP-21) or that “I don’t think you should be doing that” (FR-14). One man talked about his wife fussing over him: “she [wife] is the one who complains because she has to always be aware that I can’t do something” (FR-20). Another woman told us, “my husband will say, don’t run, because he doesn’t want me to break an ankle or something” (AD-23). Another participant joked about her children talking about her behind her back, “and you still think she’s young and she isn’t. She’s 70” (AD-5). Another woman said her daughter kept an eye out for her because she wanted “to keep me around much longer” (I-24).

A few participants talked about their friends reinforcing the message of being old. For example, one told us about her Saturday morning coffee dates with friends, “half of them take calcium. I have heard the Fosamax stories. That’s what women our age talk about. Very boring” (I-18).

Two participants appeared to not fit with our general findings that experiences after a fracture made them feel old. One participant said, “I don’t think I’m elderly” (COM-14) and another said, “I do feel that your age shouldn’t matter. You should have good bone health no matter how old you are” (I-1).

## Discussion

In this study, we demonstrated that individuals not only reported *feeling* old after sustaining a fracture, but they also appeared to internalize this feeling to the extent that their conversations reflected a resignation to *being* old. The fracture event, and the fall that led to the fracture, may not have been associated with old age and fragility as previously reported [6] but the event then triggered perceptions of subsequent experiences that made individuals *become* older. Participants’ reports of feeling old post-fracture reflect the work of Kleinman [27] who posited an explanatory model of people’s experiences of illness that are socially and culturally situated. According to Kleinman, we gather and interpret information regarding our health and illness through overlapping sectors of health care, such as the popular sector that surrounds us with information from family, friends and the media. For example, participants in our study talked about becoming their parents. For these participants, fractures did not appear to be conceptualized as an isolated event but rather a marker for the passage from being an adult to being old.

According to Robertson, the age medical practitioners deemed an individual to be old was 77 [28]. In our study, the age of participants was 45 + years of age, suggesting that messages about aging from health care providers may extend to individuals who are less than 77 years old. Future research is needed to examine how health care providers subjectively determine old age and whether ageist attitudes may be a barrier to providing appropriate care for individuals at risk for future fracture.

Participants in our study also described feeling weak and frail, mourning the loss of youth, and seeing the future as foreboding. Bruun-Olsen and colleagues similarly reported that individuals with a hip fracture felt they did not have hopes for the future and were unable to return to their former life [29]. Negative perceptions about the future may also reflect a lack of understanding about treatment options to improve bone health and decrease the risk of future fractures. Despite being screened through a fracture prevention program and receiving education about bone health management, patients have reported uncertainty about treatment [30]. More effort is needed to convey to patients that pharmacological treatment can reduce the risk of future fractures [31].

Participants’ explanatory models of aging were reinforced by health care providers. In Kleinman’s work [27], the professional sector is one facet of information used in explaining health and aging. Messages that bones were deteriorating, that healing would take longer, that certain activities were no longer appropriate and that medications were necessary wrapped participants in a context of aging. This is consistent with one review where participants reported that their physicians felt osteoporosis in general should be accepted as a normal part of aging [32]. While these messages may be supported by evidence, how this information is relayed in the clinical encounter could be re-framed. For example, we acknowledge that molecular processes are impaired in older persons [33–36]. However, clinical outcomes may also depend on the perceptions of patients [10–13]. Thus, conveying excessively pessimistic predictions of post-fracture outcomes to patients, especially if evidence of delayed age-related bone healing of some fragility fractures is lacking, may promote poor clinical outcomes.

Our results are important because perceived age discrimination has been shown to influence how old a person feels [37]. This is potentially harmful as cultural stereotypes about age are absorbed and adopted by individuals, which in turn may affect the individual’s actual aging process [38]. These results have implications for health care providers. We recommend that health care providers engage patients in discussions about their bone health, taking the time to hear and acknowledge patients’ perspectives in their social and cultural setting. While bone age may be a reassuring physiological measure for children’s skeletal development, it appeared to be especially distressing to our fragility fracture patients and significantly affected their sense of self and subjective age. Rather, we suggest that providers emphasize what can be done to improve bone health so individuals can participate in daily activities. This is aligned with the World Health Organization’s vision of active aging, a process that involves engagement in social, economic, cultural, spiritual and civic affairs in order to realize physical, social, and mental well-being throughout the life course [39]. Higher self-ratings of active aging have been shown to be correlated with higher self-ratings of health and quality of life [40]. Further, when discussing age with patients, health care providers could point out that getting older is not always perceived as a “bad” thing. While aging has been written about as a gradual loss of the body’s battle against molecular and cellular damage and degradation [41, 42], people also accumulate emotional wisdom as they age that leads them to selecting more emotionally satisfying events, friendships, and experiences [43]. Menkin and colleagues [44] showed that individuals who had more positive expectations about aging made more new friends two years later than those with negative expectations about aging.

Our findings have implications for individuals who sustain a fragility fracture. According to stereotype embodiment theory, when people internalize negative age stereotypes, this influences how they actually age [45]. If individuals resign themselves to the idea that they are just “getting old”, this perception may imply that nothing can be done about bone health. By internalizing thoughts of being old, over time, individuals may start to live like an older person where they are afraid to participate in recommended activities because of fear of falling. They may stop adhering to recommendations for calcium, vitamin D, and medication because they lack confidence that these health-promoting behaviours will maintain or improve their current bone health status. They may also withdraw from participating in physical activities that improve bone density and strength and social activities that are important for healthy aging. Levy and Meyers [46] reported that individuals who internalize negative age stereotypes are more likely to view healthy practices as futile while individuals who internalize more positive age stereotypes are more likely to engage in healthy practices such as regularly taking prescribed medication.

There are strengths and limitations to report. In secondary analyses, the quality of the data are critical [47]. The first author was lead researcher in the primary studies, so we are confident in the quality of the original data. Our analysis complements previous work demonstrating that patients become vigilant [8] and experience a need for informal care after a fragility fracture [7]. The secondary analysis design limited the current study in that we did not formulate a research question and design specific methods to address that question [47]. Consequently, we were unable to refine the methods in an iterative manner based on preliminary feedback during data collection and the development of concepts [47] as is customary with emergent analysis in qualitative research [48]. Further, the aim of the primary studies was not to answer our objective and so, our data are limited by this. We acknowledge the inherent subjective nature of our interpretations. However, we propose that our study underestimates the impact of the fracture on participants’ perceptions of becoming old because we excluded discussions about increased vigilance related to fear of falling and an increased dependence on others over time post-fracture.

## Conclusions

Individuals who sustained a fragility fracture and were at risk for subsequent factures described experiences of feeling old. This perception was reinforced by health care providers, family, and friends. Careful consideration of how bone health messages are communicated to patients post-fracture by health care providers is warranted. It is also important to consider the impact of feeling old on patients’ attitude to recommendations for bone health management post-fracture.

## Data Availability

The datasets generated and/or analysed during the current study are not publicly available due to participants not consenting to having their data deposited in a public dataset but are available from the corresponding author on reasonable request.
